# Ventilation Pathways of Middle Ear and Mastoid in Patients Undergoing Endoscopic Tympanoplasty at a Tertiary Care Center in Uttarakhand: An Observational Study

**DOI:** 10.7759/cureus.90367

**Published:** 2025-08-18

**Authors:** Mohammad Mohsin Raza, Bhawana Pant, Nitin Sharma, Saeem Ahmed

**Affiliations:** 1 Department of Otolaryngology, Head and Neck Surgery, Government Doon Medical College, Dehradun, IND

**Keywords:** graft uptake, hearing loss, otitis media, tympanoplasty, ventilation pathways

## Abstract

Background

Chronic otitis media (COM) is one of the most common diseases causing hearing impairment. Tympanoplasty is the treatment of choice for patients suffering from a mucosal type of COM. The success of tympanoplasty depends on many factors, including the ventilation pathways of the middle ear and mastoid, which can be visualized using an endoscope.

Aim

The aim of the study is to evaluate the ventilation pathways of the middle ear and mastoid in patients undergoing endoscopic tympanoplasty.

Methodology

It was an observational cross-sectional study conducted in the Department of Otorhinolaryngology - Head and Neck at a tertiary care center in North India from August 2022 to January 2024. Around 64 patients aged 18-50 years, suffering from inactive mucosal type of COM, were included in the study. Preoperative hearing assessment was performed. All patients underwent endoscopic tympanoplasty under general anesthesia. Intraoperative visualization and documentation of the ventilatory pathways of the middle ear and mastoid were performed using 0-degree and angled endoscopes. The patients were evaluated for the outcome of surgery at 4 weeks and 12 weeks in terms of graft uptake and postoperative hearing benefit.

Results

A total of 64 patients underwent transcanal endoscopic tympanoplasty in our study. Intraoperative visualization of ventilation pathways through rigid angled endoscope revealed that 55 patients (86 %) had clear anterior and posterior isthmus. Nine patients (14%) had blocked ventilation pathways, which were cleared during surgery. Graft uptake was successful in 63 patients (98.4%). The mean hearing threshold improvement was statistically significant.

Conclusion

Transcanal endoscopic tympanoplasty using a rigid angled endoscope has the advantage of visualizing the ventilation pathways and middle ear spaces through a single approach, avoiding mastoid exploration. Evaluation of the middle ear ventilation pathways during tympanoplasty can give good surgical results in terms of graft uptake and hearing improvement, as it ensures good ventilation between various compartments of the middle ear cleft.

## Introduction

Chronic otitis media (COM) is one of the most common diseases causing hearing impairment. It is divided into two categories, i.e., mucosal (safe variety) and squamous (unsafe variety). Tympanoplasty is the treatment of choice for patients suffering from a mucosal type of COM. Prerequisites of a patient undergoing tympanoplasty include a dry ear, a well-functioning Eustachian tube (ET), and good cochlear reserve [1,2]. The success of tympanoplasty depends on many factors, which include patient factors and disease factors. Tympanoplasty can be performed using both a microscope and an endoscope. The use of an endoscope in middle ear surgery provides an opportunity to visualize a detailed anatomy of all the middle ear spaces with the help of angled endoscopes (30, 45, and 70 degrees). Earlier, it was thought that in a patient with a well-functioning ET, there would be adequate ventilation of the mastoid air system. However, research has now proven that the anatomy of the epitympanic diaphragm and patency of the anterior and posterior isthmus also play a major role in the ventilation of the middle ear and mastoid air cell system [3,4]. Aeration pathway from the ET leads directly to the mesotympanic and hypotympanic spaces, whereas the epitympanum is set apart from the direct air stream and is only aerated through the tympanic isthmus [5]. Even in the presence of a well-functioning ET, a blocked isthmus tympanicum may hinder ventilation and pneumatization of the atticomastoid compartment of the middle ear cleft, resulting in sclerosed mastoid and possible formation of attic retraction and cholesteatoma [3]. Postoperative hearing benefits may be affected by inadequate functioning of the ventilatory pathways of the middle ear and mastoid in the presence of underlying chronic infection in the middle ear [6]. Not much work has been carried out in this regard in the past, so this study aims to evaluate the ventilation pathways of the middle ear and mastoid intraoperatively during endoscopic tympanoplasty and its significance in terms of predicting postoperative graft uptake and hearing benefits. This study received ethical clearance from the institutional ethics committee. 

## Materials and methods

An observational cross-sectional study was conducted in the Department of Otorhinolaryngology and Head and Neck at Government Doon Medical College and Hospital, Dehradun, Uttarakhand, a tertiary care center in North India. The period of study was from August 2022 to January 2024 (18 months). A total of 64 patients aged 18-50 years, suffering from inactive mucosal type of COM with normal ET function, were included in the study. Patients with revision surgery, sinonasal pathology, and systemic disorders such as diabetes mellitus were excluded from the study. Each patient was evaluated in detail through a proforma designed for history and clinical examination. Otoendoscopic examination was performed in each patient, and findings were documented by an endoscopic camera system (Image-S) with a high definition monitor and recording facility from Karl Storz, Tuttlingen, Germany. Preoperative hearing assessment was performed using pure tone audiometry (PTA) (Interacoustic AC-40), otoacoustic emission, and impedance audiometry (Interacoustic AT-235). Digital X-ray mastoid (Schuller’s view) was performed in each patient to assess the mastoid air cell pneumatization. All patients underwent endoscopic tympanoplasty under hypotensive general anesthesia. Intraoperative visualization and documentation of the ventilatory pathways of the middle ear and mastoid were done using zero-degree and angled rigid endoscopes (30, 45, and 70 degrees). Blocked pathways were opened. The patients were followed up at the 10th day, 4 weeks, and 12 weeks after surgery. Postoperative status of hearing was assessed by PTA at 4 and 12 weeks. 

Operative steps 

**Figure 1:**
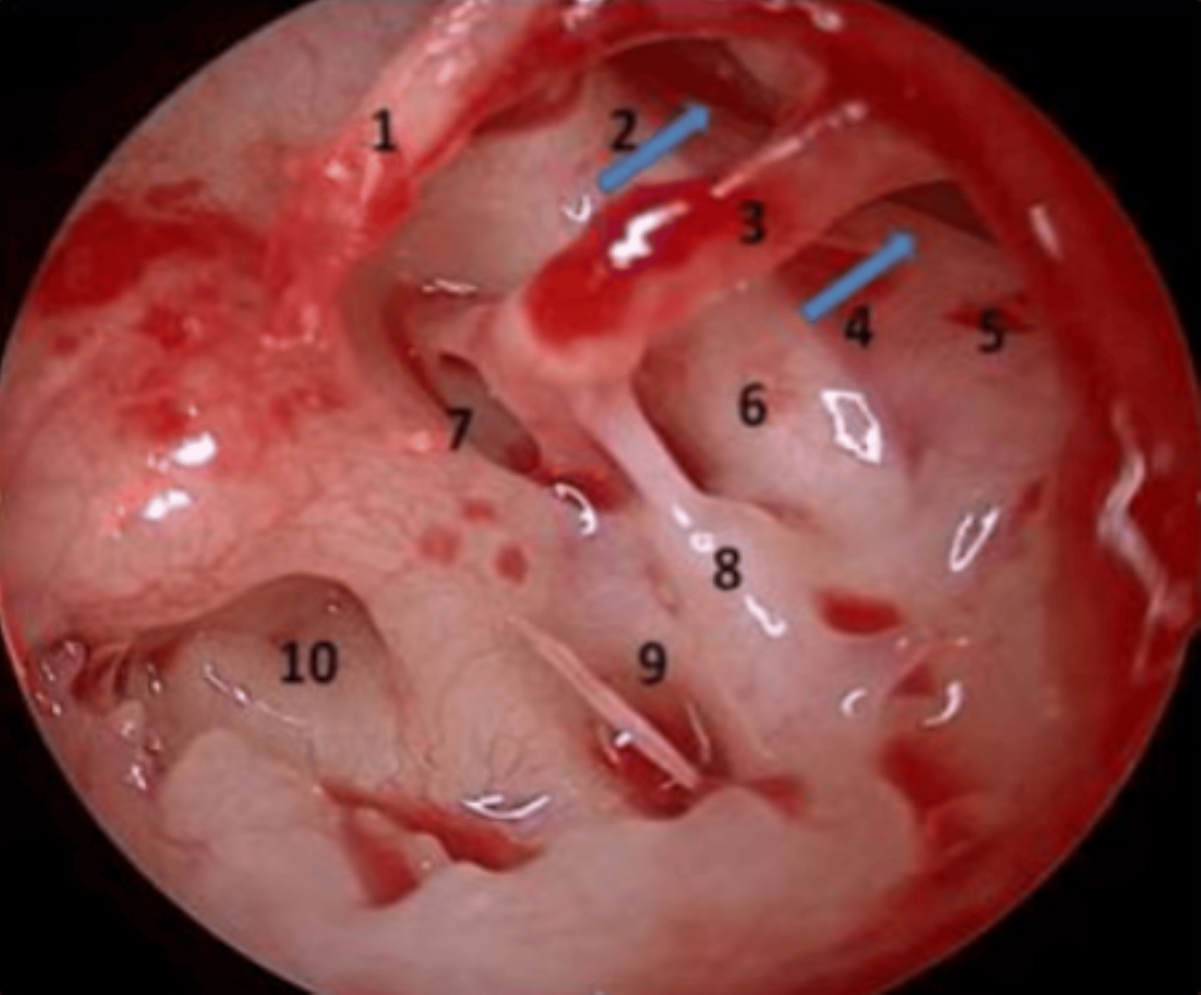
Intraoperative view of the ventilation pathways using an angled endoscope (left ear) after elevation of the tympanomeatal flap. (1) Maleus; (2) anterior isthmus (arrow); (3) incus; (4) posterior isthmus (arrow); (5) lateral semicircular canal; (6) fallopian canal; (7) oval window; (8) pyramidal eminence; (9) sinus tympani; (10) round window. Blue arrows indicate anterior isthmus (number 2) and posterior isthmus (number 4).

**Figure 2:**
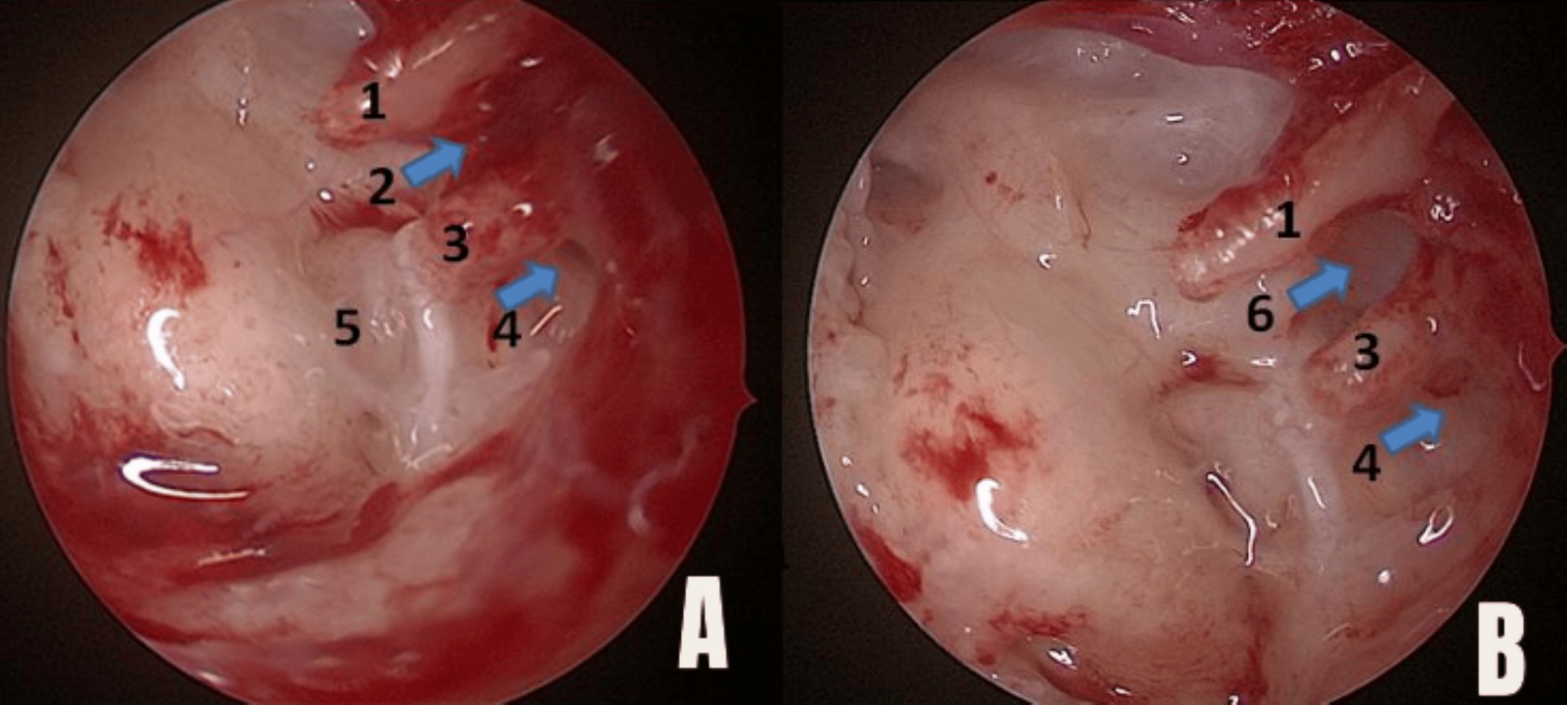
Intraoperative view of the ventilation pathways using an angled endoscope (left ear) after elevation of the tympanomeatal flap. (1) Maleus; (2) anterior isthmus (arrow); (3) incus; (4) posterior isthmus; (5) oval window; (6) mucosal fold is removed and patency of the anterior isthmus established (arrow). (A) Before removing the mucosal fold from the anterior isthmus; (B) after removing the mucosal fold from the anterior isthmus. Blue arrows indicate blocked anterior isthmus (number 2), posterior isthmus (number 4), and patent anterior isthmus after removal of the mucosal fold (number 6).

**Figure 3:**
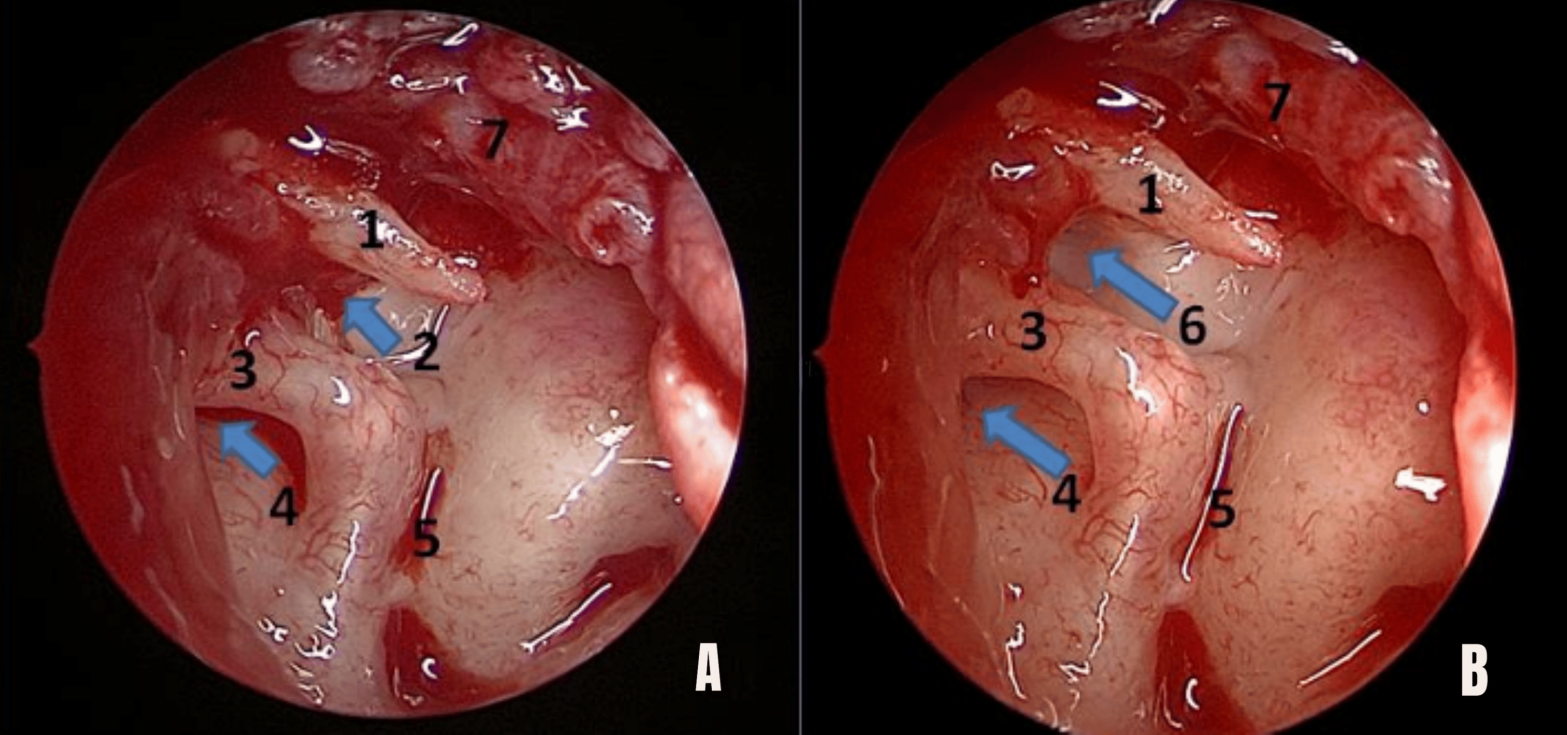
Intraoperative view of a blocked ventilation pathway with an angled scope (right ear) after elevation of the tympanomeatal flap. (1) Malleus; (2) anterior isthmus blocked with a mucosal fold (arrow); (3) incus; (4) posterior isthmus (arrow); (5) oval window; (6) mucosal fold removed and patency of the anterior isthmus established (arrow); (7) tympanomeatal flap. (A) Before removing the mucosal fold from the anterior isthmus; (B) after removing the mucosal fold from the anterior isthmus. Blue arrows indicate blocked anterior isthmus (number 2), posterior isthmus (number 4), and patent anterior isthmus after removal of the mucosal fold (number 6).

All patients underwent endoscopic transcanal tympanoplasty under general anesthesia. Routine instruments for micro ear surgery were used. The temporalis fascia was harvested for graft. The perforation was localized, and the margins of the perforation freshened. Radial incisions were made at 6 o’clock and 12 o’clock positions from lateral to medial direction using Plester’s knife, and both incisions were connected using Rosen’s knife. The flap was elevated, and the middle ear was entered without injuring the chorda tympani. The handle of the malleus was skeletonized. The status of the ossicles and their mobility was checked by eliciting the round window reflex. Anterior and posterior isthmus were visualized (Figure 1). If any blockage was found, it was removed (Figures 2, 3). The ET opening was visualized in each patient, which was normal in appearance using an angled scope. No inflammatory tissue was observed obstructing the ET opening. The findings were noted. The graft was placed via an underlay technique with the placement of pieces of Gelfoam in the middle ear. The ear canal was packed with ointment-soaked gauze tape. 

Postoperative care 

All patients were administered prophylactic antibiotic (inj. ceftriaxone 1 gm IV), xylometazoline nasal drops thrice a day, tab cetrizine 10 mg once a day, and tab aceclofenac (100 mg twice daily) in the postoperative period. The patient was instructed not to sniff. All the patients were discharged on the second postoperative day on oral antibiotic tab amoxycillin and clavulanic acid 625 mg twice a day and application of topical mupirocin ointment over the incision site used for harvesting the temporalis fascia graft. The patients were reviewed on the 10th postoperative day for removal of the ear pack and suture removal. Patients were advised to keep their ears dry and abstain from forceful nose blowing, exposure to cold, and swimming.

Follow-up 

All the patients were followed up on the 10th day, 4 weeks, and 12 weeks after surgery. Medical management was performed accordingly, and documentation was carried out in each follow-up visit. The patient’s hearing was evaluated at 4 weeks and 12 weeks using PTA at speech frequencies of 500, 1000, 2000, and 4000 Hz. The postoperative audiogram was compared with preoperative finding.

Statistical analysis

In the analysis of qualitative data, a non-parametric test was employed. For quantitative data, a parametric test was employed. The data were analyzed with the Statistical Package for Social Sciences (SPSS) version 22 (IBM Corp., Armonk, NY). The p-value of <0.05 was considered significant.

## Results

Demographic characteristics

The majority of patients (42 out of 64, i.e., 65.6%) belonged to the age group of 18-30 years. Out of 64 patients, 47 were female and 17 were male. The female-to-male ratio was 2.76:1. Thirty-six patients (59.2%) belonged to the lower middle class. Sixteen (25%) patients had bilateral disease, while the rest had unilateral disease.

Clinical features

Discharge from the affected ear and hearing loss were the main complaints in all the patients, while only two patients had no complaint of any hearing loss. The mean duration of discharge in our subjects was 4.6 years. The majority of patients, i.e., 34 out of 64 (53.1%), had large central perforation. 

Mastoid pneumatization

X-ray mastoid (Schuller’s view) was performed in all the patients. Pneumatization of mastoid air cells was observed in each case, as shown in Table 1. Around 33 (51.6%) patients had partially pneumatized (diploic) mastoid air cells, 20 (31.2%) patients had poor pneumatization of mastoid air cells (sclerotic), and 11 (17.2%) patients had well pneumatized mastoid air cells.

**Table 1:** Showing pneumatization pattern of mastoid air cells

Pneumatization pattern of mastoid air cells on X-ray	Frequency	Percentage
Partially pneumatized (diploic)	33	51.6%
Poorly pneumatized (sclerosed)	20	31.2%
Well pneumatized	11	17.2%
Total	64	100%

Ventilation pathways

A total of 64 patients underwent transcanal endoscopic tympanoplasty in our study. Intraoperative visualization of ventilation pathways through rigid angled endoscope revealed that 55 patients (86%) had clear anterior and posterior isthmus. Nine patients (14%) had blocked ventilation pathways, which were cleared during surgery, as shown in Table 2.

**Table 2:** Showing status of ventilation pathways

Anterior and posterior isthmus	Frequency	Percentage
Clear	55	86%
Blocked	9	14%
Total	64	100%

Postoperative graft uptake

At the end of three months, 63 out of 64 patients (98.4%) had successful graft uptake. Only one patient (1.6%) had graft failure, as shown in Table 3.

**Table 3:** Showing graft uptake after three months of surgery

Graft uptake	Frequency	Percentage
Yes	63	98.4%
No	1	1.6%
Total	64	100%

Preoperative vs. postoperative PTA threshold

The mean hearing threshold improved from 43.45 dB at the preoperative time to 26.88 dB three months after surgery. This change was statistically significant (Wilcoxon test: V=2009.5, p=<0.001), as shown in Table 4.

**Table 4:** Showing difference between preoperative and postoperative hearing threshold dB: decibels; IQR: Interquartile range; SD: Standard deviation; PTA: Pure tone audiometry

Time point	PTA (hearing threshold) (dB)			Wilcoxon test	
	Mean (SD)	Median (IQR)	Range	V	p-value
Preoperative	43.45 (10.49)	43.50 (15.00)	26.00 to 73.00	2009.5	<0.001
Postoperative	26.88 (10.65)	25.00 (10.00)	14.00 to 70.00		
Absolute change	-16.58 (8.10)	-16.00 (9.25)	-32.00 to -3.00		
Percent change	-38.2% (16.2)	-41.7% (19.1)	-64% to -9%		

Preoperative vs. postoperative air bone gap

The mean air bone gap decreased from 28.67 dB at the preoperative timepoint to 13.28 dB 3 months postoperatively. This change was statistically significant (Wilcoxon test: V=1889.5, p=<0.001), as shown in Table 5. 

**Table 5:** Showing difference between preoperative and postoperative air bone gap B: decibels; IQR: Interquartile range; SD: Standard deviation

Time point	Air bone gap (dB)			Wilcoxon test	
	Mean (SD)	Median (IQR)	Range	V	p-value
Preoperative	28.67 (8.15)	26.00 (11.25)	15.00 to 46.00	1889.5	<0.001
Postoperative	13.28 (6.86)	12.00 (8.25)	6.00 to 40.00		
Absolute change	-15.39 (7.96)	-15.00 (9.50)	-32.00 to -3.00		
Percent change	-52.8% (21.6)	-60.0% (19.7)	-84% to -13%		

## Discussion

Surgical management of COM (mucosal) has evolved in recent times in order to give better outcomes in terms of graft uptake and hearing benefit. Factors favorable for a good outcome after tympanoplasty include a dry ear, well-functioning ET, and good cochlear reserve [1]. However, other factors also play a role in the postoperative outcomes, including patient factors (hygiene, immunity, etc.) and middle ear compliance, which in turn depend upon ventilatory pathways of the middle ear.

ET plays an important role in maintaining middle ear pressure and the clearance of middle ear secretions into the nasopharynx. Any dysfunction in ET can adversely affect the success rate of tympanoplasty [3]. The ET function test is performed preoperatively to know the status of ET functioning and middle ear compliance. Clinically, it is assessed by the Valsalva maneuver, where it is ascertained whether an artificially increased or decreased middle ear pressure can be neutralized and brought back to normal pressure by swallowing. The ET function by the impedance audiometer assesses the physiological function of ET, which is more important to the clinician than the mere assessment of the anatomical patency of the tube. William’s test is performed in subjects with an intact tympanic membrane, and Toynbee’s test is the test of tubal function in subjects with a perforated membrane [7]. The pathogenesis of COM has been related to the presence of nasal, paranasal, or nasopharyngeal diseases, which can obstruct the ET, leading to the development of COM [8]. The graft uptake was found to be high in patients with visualization of normal ET opening on nasal endoscopy in some studies [8,9].

The aeration of the middle ear occurs through two pathways: anteriorly via the ET, which directly ventilates the mesotympanum and hypotympanum. The aeration of epitympanum depends on the anterior and posterior isthmus and an incomplete tensor fold [10]. The visualization of the ventilation pathways is difficult with a microscope during middle ear surgeries. However, the rigid angled endoscopes (30, 45, and 70 degrees) give a better view of the ventilation pathways during middle ear surgeries and, if blocked, can be cleared during surgery [11]. Marchioni et al. studied the middle ear ventilation route blockage in 22 participants suffering from cholesteatoma. Fourteen patients out of 22 had a blocked isthmus. The authors endorsed the use of an endoscope during middle ear surgery to address the ventilation pathways [5]. Hazarika et al., in a study on 157 patients of mucosal type chronic suppurative otitis media and squamous type with attic cholesteatoma, used an angled endoscope to visualize the anterior and posterior isthmus and the tensor fold. Any disease process (granulation or mucosal edema) found was removed, and ventilation was reestablished. This significantly improved outcome after surgery [12].

It has been observed that a poorly ventilated mastoid may also adversely affect the outcome of tympanoplasty in patients with a mucosal type of COM [4]. Pneumatization pattern of mastoid air cells gives an idea about the status of mastoid ventilation, which in turn determines the success of surgery in terms of graft uptake and hearing improvement. Metin et al. found in their study that the graft uptake was better in the well-ventilated mastoid group as compared to the poorly ventilated group, but it was not statistically significant [13]. Homquist and Bergstrom measured the mastoid volumes using Schuller X-rays performed preoperatively and demonstrated that the middle ear retraction was greater in patients who had undergone tympanomastoidectomy than those who had undergone tympanoplasty without mastoidectomy. They advocated that it is better not to explore a well-ventilated mastoid during middle ear surgery [14]. 

Marchioni et al. described blockage of the middle ear ventilation pathway during endoscopic ear surgery and observed the preoperative mastoid pneumatization using a CT scan. They found that out of 22 patients with a blocked ventilation pathway, 18 patients had hypo-pneumatized or sclerosed mastoid. Results were statistically significant [5]. In our study, it was observed that poorly pneumatized mastoid air cells were more commonly associated with blockage of the ventilation pathway than well pneumatized mastoid air cells. The success rate of surgery in terms of graft uptake in our study was 98.4%. This could be due to the fact that ventilation pathways were addressed in each patient and cleared if found blocked. 

In our study, the mean hearing threshold improved from 43.45 dB at the preoperative time to 26.88 dB three months after surgery. The mean air bone gap decreased from 28.67 dB preoperatively to 13.28 dB three months after surgery. The changes were statistically significant. Indorewala et al. [15], Olusesi et al. [16], and Ogisi et al. [17] found similar hearing results in their studies. Studies have been conducted to evaluate the other ventilatory pathways of the middle ear apart from the anterior and posterior isthmus, which might adversely affect the outcome of middle ear surgery. The aditus ad antrum and the tensor fold (complete or incomplete) also play a role in the ventilation pathway, which has been studied in the past in patients suffering from mucosal and squamous COM [5,12].

Although few studies are addressing the role of ventilation pathways in middle ear surgery, studies with larger sample sizes are required. Not everyone is well-versed in endoscopic surgery, so it requires more training. Also, smaller endoscopes are needed for narrow ear canals, which are not available at every center.

## Conclusions

Tympanoplasty is the surgery advised for patients with a mucosal type of COM. Transcanal endoscopic tympanoplasty using a rigid angled endoscope has the advantage of visualizing the ventilation pathways and middle ear spaces through a single approach, avoiding mastoid exploration. The endoscope also provides the advantage of less surgical trauma and reduced postoperative pain. Patent ventilation pathways can give good surgical results in terms of graft uptake and hearing improvement, as they ensure good ventilation between various compartments of the middle ear cleft. 
